# Linking Environments and Resident Function via MDS

**DOI:** 10.1093/geroni/igaf122.622

**Published:** 2025-12-31

**Authors:** Migette Kaup, Adam Davey, Margaret Calkins, Robert Wrublowsky

**Affiliations:** Kansas State University, Manhattan, Kansas, United States; University of Delaware, Newark, Delaware, United States; IDEAS Institute, Cleveland Heights, Ohio, United States; RAW Consulting, Winnipeg, Manitoba, Canada

## Abstract

Over the past three decades, research in long-term care settings has demonstrated links between design characteristics and the experiences of residents who live in these environments. Individual studies have shown how different approaches to the design of the built environment can contribute to enhanced resident safety, function, and even care outcomes but these findings have been difficult to generalize due to the lack of a validated and comprehensive assessment tool that provides a consistent metric for the discrete environmental characteristics that may be contributing factors. In addition, existing environmental tools have limited demonstrated connections to other widely used assessment tools for care outcomes such as the Minimum Data Set (MDS) the standard for facilitating care management in nursing homes. The Environmental Audit Scoring Evaluation (EASE) tool has been shown to provide a reliable set of comprehensive scoring criteria that can measure the strengths (or weaknesses) of specific design features. Further, the EASE tool items are comprised of features that specifically support quality outcomes measures that are also of focus of the MDS. In this paper, we describe the study design to link the MDS assessments of 235 residents to the EASE assessments of their (15) distinct living areas across seven skilled care buildings.

